# Nuclear Medicine in Sports

**DOI:** 10.4103/0972-3919.78242

**Published:** 2010

**Authors:** Anshu Rajnish Sharma

**Affiliations:** Editor-in-Chief, IJNM; Consultant, Nuclear Medicine and PET, Kokilaben Dhiurubhai Ambani Hospital and Medical Research Institute, Four Bunglows, Andheri (W), Mumbai, India. E-mail: anshurajneesh@rediffmail.com

The recent triumphs of our sportspersons in the Common Wealth Games, Asian Games, Cricket, and other sports have made us believe that we are changing from a ‘Sports Loving’ to a ‘Sporting’ nation.

Sports has become a serious profession in the era of advertising and marketing. It is no more a leisure or relaxing activity. Sportsmen are under tremendous pressure from passionate fans and brand sponsors to deliver the best performance, at the highest level of competition. Sportsmen are actively engaged in physical workouts, not to keep themselves physically fit, but rather to enhance their physical stamina and endurance. This vigorous physical activity may at times inflict overuse musculoskeletal injuries on sportsmen, which may risk their professional career at its peak.

We need to recognize this huge responsibility and prepare ourselves to take care of our beloved star performers and diagnose their sports-related stress injuries at an early stage. This will help in shortening the inactivity period, preventing further aggravation of the injury, and early rehabilitation of the sportsmen.

Bone stress injuries account for about 10% of the sports medicine practice. Female athletes run three to four times higher risk of bone stress injuries as compared to their male counterparts. Lower extremity bones are most commonly affected (70 – 95%) followed by the spine. The tibia is the most common lower extremity bone reported / known to have stress fractures (50%), followed by the metatarsal, navicular, femur, fibula, and sesamoid bone. Stress injuries to the spine (pars inter-articularis) commonly affect adolescent and young athletes (up to 15%). Fast bowlers in cricket are more prone to have stress injuries of the lumbar spine. At a given time, 50% of the fast bowlers of a national team remain out of action due to stress injuries.

Nuclear Medicine can synergistically contribute to the Sports Medicine field, in the management of sports-related stress injures. Bone Scintigraphy is commonly requested for evaluation of athletes with pain. Three-Phase Tc-99m MDP Bone Scan has emerged as the imaging reference standard for diagnosing such injuries. The inherently high-contrast resolution of the bone scan allows early detection of bone trauma and becomes positive within six to seventy-two hours after the onset of symptoms. The bone scan is able to demonstrate stress injuries days to weeks before the radiograph. In the initial stages, X-rays may look normal *(low sensitivity 15 – 35%)* and turn positive in 30 – 70% of the cases on follow-up. However, still, the X-ray remains the primary / first imaging tool for excluding other differentials *(chronic sclerosing osteomyelitis, Ewings’ sarcoma, Osteoid Osteoma)*. If the X-ray is unremarkable with a clinical suspicion of bone stress injury, then the patient should be subjected to a three-phase bone scan. If a three-phase bone scan is negative, bone pathology (stress injury) is quite unlikely. The bone scan is known for its high sensitivity equaling that of a magnetic resonance imaging (MRI) scan. However, there are few case reports, where a bone scan has been found to be false negative at initial presentation, but became positive on repeat study. The bone scan, because of its ability of whole body imaging, detects unsuspected multi-focal trauma or abnormality that is away from the symptomatic site. Various parameters like intensity and location of tracer uptake and extent of involvement (thickness) of bone, provide useful prognostic information about the severity of the injury, period of inactivity, duration of resumption of rehabilitation, and high risk of developing complications like complete fracture, non-union, or osteonecrosis. Zwas *et al*. have suggested a classification system for grading the bone stress injuries (Grade I – IV) based on a scintigrapic pattern way back in 1988. Grades I and II injuries are mild grade take a shorter period for healing, and allow an early return to activity, as compared to the higher grade (III and IV) injuries. Location of uptake (injury) involving the tensile aspect (superior) of the femoral neck, anterior cortex of the tibia, and the fifth metatarsals and sesamoid bone are high risk.

Bone *single photon emission computed tomography* (SPECT) and SPECT-CT have high diagnostic accuracy in the precise localization of acute stress reaction and stress fractures involving the posterior elements of the lumbar spine (pedicle, lamina / pars-interarticularis). Stress fractures will show a focal abnormal uptake corresponding to the pars-defect (spondylolysis) seen on the computed tomography (CT) component of the SPECT-CT, whereas, the focal uptake in the pars / pedicle of the lumbar spine, without any morphological abnormality, is consistent with a stress reaction (pre-fracture phase), and ascertains the reason for the recent back pain in an athlete.

Similar to the Tc-99m MDP Bone SPECT, the F-18 NaF Skeletal PET-CT also shows focal metabolic abnormality at the site of the stress, secondary to sports activity. There are few recent publications describing the utility of the F-18 NaF Skeletal PET-CT for this very clinical indication. Fluoride Skeletal PET has higher spatial resolution and sensitivity, and thus provides superior image quality as compared to the Tc-99m MDP bone scan. The F-18 NaF Skeletal PET-CT is now regarded as the new-age bone scan. Problem of limited specificity is circumvented by the CT component of 18F- NaF PET-CT.

Magnetic resonance imaging is the imaging modality of choice for stress injuries. However, with the advent of PET-MRI scanners and whole body imaging capability being practically feasible, the PET-MRI may become a one-stop solution, in future, for a wide spectrum of musculoskeletal disorders, including sports-related stress injuries.

